# Prospective randomized controlled pilot study on the effects of almond consumption on skin lipids and wrinkles

**DOI:** 10.1002/ptr.6495

**Published:** 2019-10-01

**Authors:** Negar Foolad, Alexandra R. Vaughn, Iryna Rybak, Waqas A. Burney, Gwen M. Chodur, John W. Newman, Francene M. Steinberg, Raja K. Sivamani

**Affiliations:** ^1^ School of Medicine, University of California Davis California; ^2^ Department of Dermatology University of California Davis California; ^3^ College of Medicine, Drexel University Philadelphia Pennsylvania; ^4^ Department of Nutrition and Graduate Group in Nutritional Biology University of California Davis California; ^5^ United States Department of Agriculture Agricultural Research Service, Western Human Nutrition Research Center Davis California; ^6^ Department of Biological Sciences California State University Sacramento California; ^7^ Pacific Skin Institute Sacramento California

**Keywords:** almonds, fatty acids, lipids, skin barrier, skin physiology/structure, wrinkles

## Abstract

**Objective:**

Almonds are a rich source of fatty acids and antioxidants, and their supplementation is known to significantly modulate serum lipids. The effects of almond on the skin's lipid barrier and the appearance of wrinkles have not yet been elucidated. The aim of this study was to investigate the effects of almond consumption on facial sebum production and wrinkles.

**Methods:**

This was a prospective, investigator‐blinded, randomized controlled trial in which subjects consumed 20% of their daily energy consumption in either almonds or a calorie‐matched snack for 16 weeks. This study was completed at the UC Davis Dermatology clinic. Participants were a volunteer sample of generally healthy postmenopausal females with Fitzpatrick skin types 1 and 2. A facial photograph and image analysis system was used to obtain standardized photographs and information on wrinkle width and severity at 0, 8, and 16 weeks. Measurements of transepidermal water loss and sebum production were also completed at 0, 8, and 16 weeks.

**Results:**

Fifty healthy postmenopausal females were recruited, 31 participants were enrolled, and 28 completed the study. Under photographic analysis, the almond group had significantly decreased wrinkle severity and width compared with the control group at 16 weeks (*p* < .02). Changes in skin barrier function were nonsignificant, measured by the transepidermal water loss (*p* = .65) between the almond and control groups relative to baseline after 16 weeks. No adverse effects were reported.

**Conclusion:**

Our study demonstrates that daily almond consumption may reduce wrinkle severity in postmenopausal females to potentially have natural antiaging benefits.

## 1 INTRODUCTION

Skin wrinkling results over time from intrinsic aging processes (e.g., menopausal status) and external environmental stressors (e.g., sun exposure, cigarette smoking, and obesity) that accumulate over a lifetime (Zouboulis & Makrantonaki, 2012). Intrinsic processes associated with chronological aging of the skin lead to decreased cell and tissue regeneration and dryness, reflected by slower DNA repair in aging skin cells and reduced proliferative capacity (Gerstein, Phillips, Rogers, & Gilchrest, 1993; Ghadially, Brown, Sequeira‐Martin, Feingold, & Elias, 1995; Goukassian, Bagheri, El‐Keeb, Eller, & Gilchrest, 2002; Moriwaki, Ray, Tarone, Kraemer, & Grossman, 1996; Sauder, 1986; Sunderkotter, Kalden, & Luger, 1997). With regard to antiaging and facial skin rejuvenation, the market for topical treatments and cosmetic procedures is immense and growing exponentially, estimated to reach over $26 billion by 2021 (Chamberlain, Rhinehart, Shaefer, & Neuman, 2016). There is increasing interest by consumers and patients and growing research within dermatology that addresses both the theoretical and proven effects of various foods on skin appearance and skin aging processes.

Almonds are a rich dietary source of a range of fatty acids, polyphenols, and other phytochemicals with antioxidant properties (Alasalvar & Bolling, 2015). The modulation of serum lipid profiles by almond supplementation has been studied in detail. Conversely, their effects on the skin's lipid barrier function are under studied, although alteration of the skin barrier can improve several skin features including wrinkles (Lademann et al., 2016; Yoon et al., 2016). The sebaceous glands, housed within the dermis, are in close contact to systemic blood circulation. These glands directly communicate with the skin surface through their rich secretion of lipids (including fatty acids, ceramides, and alpha‐tocopherol) to augment the barrier and provide antioxidant properties (Ekanayake‐Mudiyanselage, Kraemer, & Thiele, 2004). Therefore, almond consumption may contribute to the photoaging defenses of the skin. The goal of this study was to understand how regular dietary intake of almonds affects facial wrinkle development.

## 2 MATERIALS AND METHODS

### 2.1 Study participants

This study was conducted from November 2016 to January 2018 as a randomized, investigator‐blinded, 16‐week clinical study. The study was approved by the Institutional Review Board at the University of California, Davis and registered on http://ClinicalTrials.gov (NCT02954315). All participants provided written informed consent prior to participation and received financial compensation. Fifty healthy females were recruited and screened for eligibility at the UC Davis Dermatology clinic. Participants were included if they were postmenopausal women with a Fitzpatrick skin type 1 or 2. Participants were excluded if they had a nut allergy, had an autoimmune photosensitive condition, or a known genetic condition with a deficiency in collagen production. Participants were also excluded if they already obtained at least 20% of their energy intake from nut consumption and those with implausible reported energy intakes of <1,000 or >3,000 kcal/day. Individuals were also excluded if they were unwilling to discontinue high‐antioxidant supplements and daily food sources, which included antioxidant supplements, beverages with added vitamins, nutrition bars with high added vitamin E, omega‐3 fatty acid supplements, herbal supplements, nuts, nut milks, or nut oil intake other than that provided by the study, sunflower seeds, and nut butters. Current tobacco smokers, those who have smoked within the past year, and former smokers with greater than a 20 pack‐year history of smoking within the past 20 years were also excluded.

### 2.2 Study interventions

The Almond Board of California provided the almonds used in this study. Almonds were provided to intervention subjects as raw almonds supplying 20% of total daily energy requirements as estimated by the Mifflin‐St Jeor equation, a validated equation that predicts caloric needs based on height, weight, age, and sex, rounded to the nearest 100 kcal (Mifflin et al., 1990). Participants received an average of 340 kcal/day of almonds (approximately 2.1 oz/58.9 g), packaged into daily portions. The control snack was commercially available, individually wrapped food products that were nut‐free and matched for calorie intake: a cereal bar, a small granola bar, and pretzels. Participants were advised to consume their provided dietary intervention (either almonds or nut‐free snack) daily and to avoid all nuts or nut‐containing products, but otherwise, to continue their normal total daily energy consumption.

### 2.3 Study design

**Figure 1:**
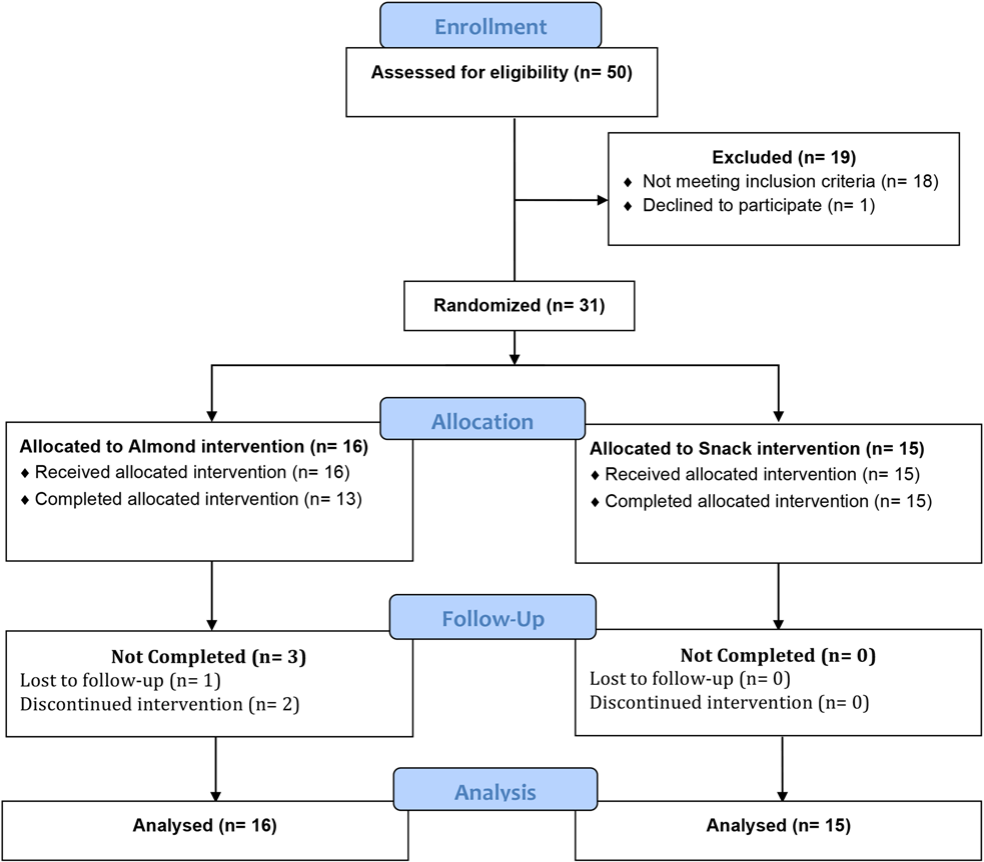
CONSORT flow diagram [Colour figure can be viewed at http://wileyonlinelibrary.com]

This 20‐week study consisted of a total of five study visits following a 4‐week dietary washout period: baseline, 4, 8, 12, and 16 weeks. Thirty‐one eligible participants (all postmenopausal females) were randomized into two intervention groups: almond group (ages 55–80 years old) and control group (ages 53–75 years old); see Figure 1 for CONSORT diagram. All treatments were prerandomized using a computer‐based randomization generator with blinded allocation through sealed envelopes. The two intervention groups consisted of those receiving almonds and those who received a nut‐free calorie‐matched snack. Participants were enrolled and assigned into one of the groups by the clinical research coordinator.

The BTBP 3D Clarity Pro® Facial Modeling and Analysis System (Brigh‐Tex BioPhotonics, San Jose, California) was utilized to obtain high‐resolution facial photographs with standardized lighting for all study participants at the baseline visit, 8 and 16 weeks. Facial wrinkle depth and severity were analyzed using previously validated 3D facial modeling and measurement (Foolad, Shi, Prakash, Kamangar, & Sivamani, 2015). The photography system utilizes three cameras and assessment of shadows for measurement of depth. The severity score is based on both the depth and the visible length of the wrinkles. The investigators assessed skin barrier function by measuring sebum production (Sebumeter® SM 815; Courage and Khazaka, Cologne, Germany) and TEWL (Vapometer; Delfin Technologies, Stamford, CT) at baseline, 8 and 16 weeks. Subjects were asked to report any adverse effects throughout the study.

### 2.4 Statistical analysis

An a priori power analysis showed that there was greater than 80% power to detect a 10% difference in wrinkle severity between the almond and control groups at Week 16, with recruitment of at least 15 subjects in each group with alpha set to.05. The investigators performed an intention to treat analysis by including all subjected that were enrolled in the trial and received any study intervention. Statistical analyses were performed using paired *t* tests (or Wilcoxon signed‐rank test for nonparametric measures) with correction for repeated measures. *P* values less than.05 and false discovery *Q* values less than 0.2 were considered significant.

## 3 RESULTS

Of the 50 subjects who were screened, 31 met enrollment criteria and were randomized into one of the two intervention groups: almond group or nut‐free, calorie‐matched snack group. Of these subjects, one subject was lost to follow‐up and unable to be contacted by the research team, and two subjects dropped out because they were unable to consistently consume the amount of almonds provided to them. All 31 subjects were included in analysis; the demographic characteristics of each group were similar (see Table 1).

**Table 1:** Demographics of subjects enrolled (includes dropouts)

Demographic factor	Almond group (*n* = 16)	Control group (*n* = 15)
Age, mean ± *SD*	63.63 ± 7.09	58.93 ± 6.10
Sex, female	16	15
Body mass index (kg/m^2^), mean ± *SD*	30.7 ± 7.31	29.7 ± 7.66

Abbreviation: *SD*, standard deviation.

### 3.1 Sebum production

**Figure 2:**
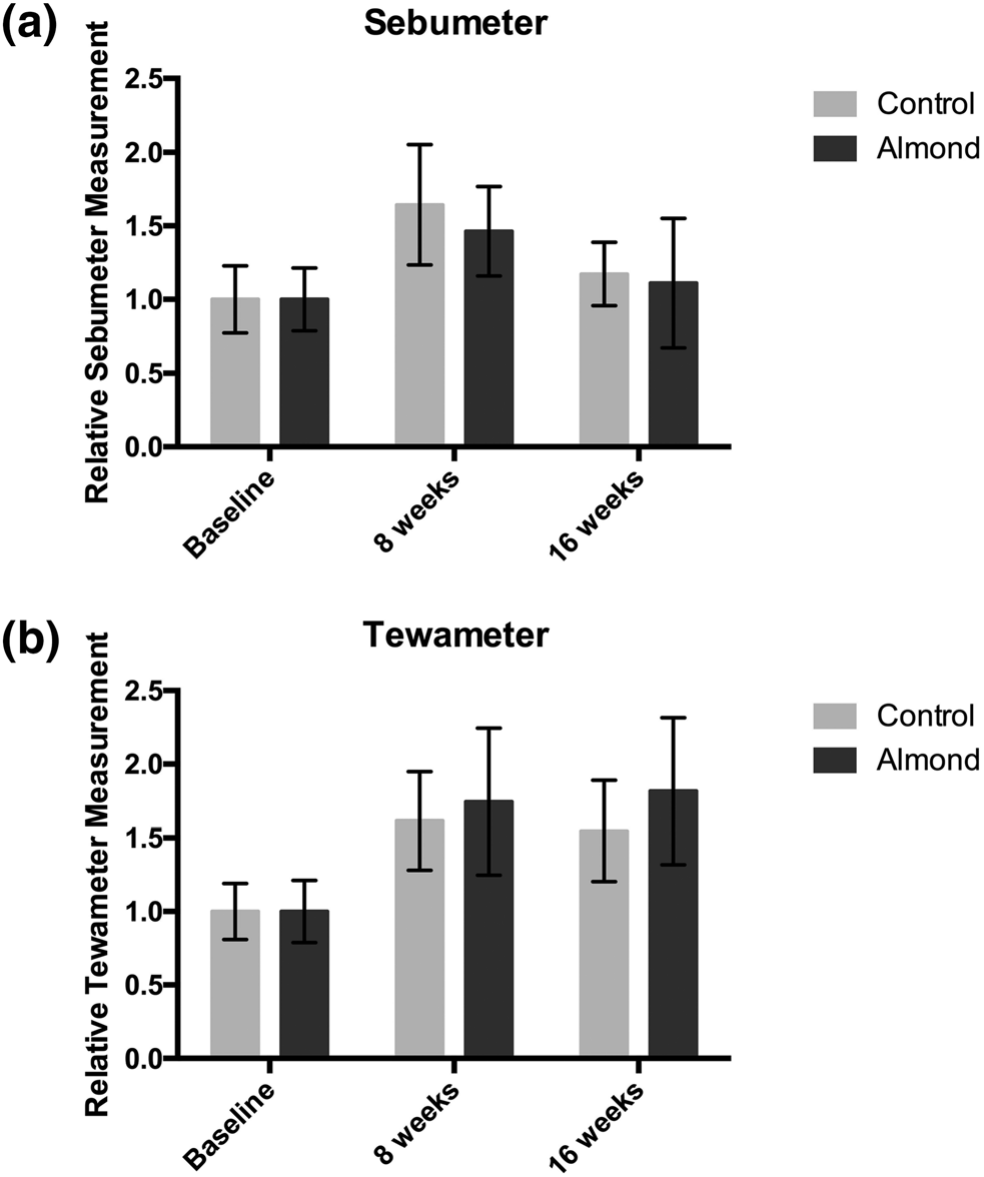
Healthy postmenopausal females were given either almonds of a calorie‐matched snack for 16 weeks. Changes in relative (a) sebum (b) transepidermal water loss were measured at baseline, 8 and 16 weeks; no significant differences were detected between the almond and control groups

There were no significant differences in sebum production between the almond and control groups after 8 (*p* = .73) or 16 weeks (*p* = .90); see Figure 2a.

### 3.2 Transepidermal water loss (TEWL)

No significant differences were detected in TEWL in the almond group versus control group relative to baseline at 8 (*p* = .82) and 16 weeks (*p* = 0.65); see Figure 2b.

### 3.3 Computer‐based photographic analysis of facial wrinkles

**Figure 3:**
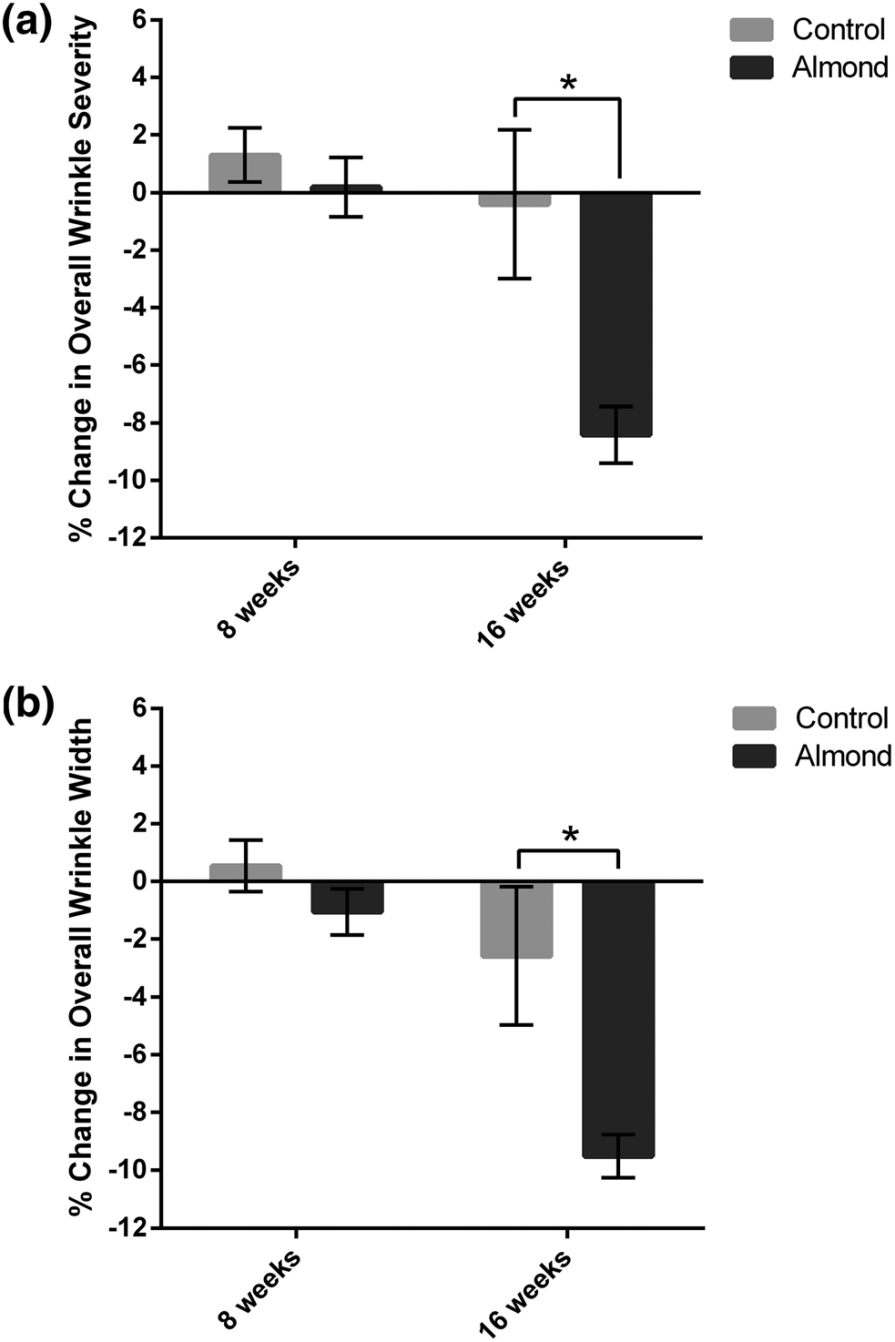
Computer‐based photographic analysis of wrinkle severity (a) and wrinkle width (b) were significantly decreased by 9% and 10%, respectively, in the almond intervention group compared with the control intervention group after 16 weeks (*p* < .02)

Wrinkle severity (Figure 3a) and wrinkle width (Figure 3b) were significantly decreased by 9% and 10%, respectively, in the almond intervention group compared with the control intervention group after 16 weeks (*p* < .02).

## 4 DISCUSSION

**Figure 4:**
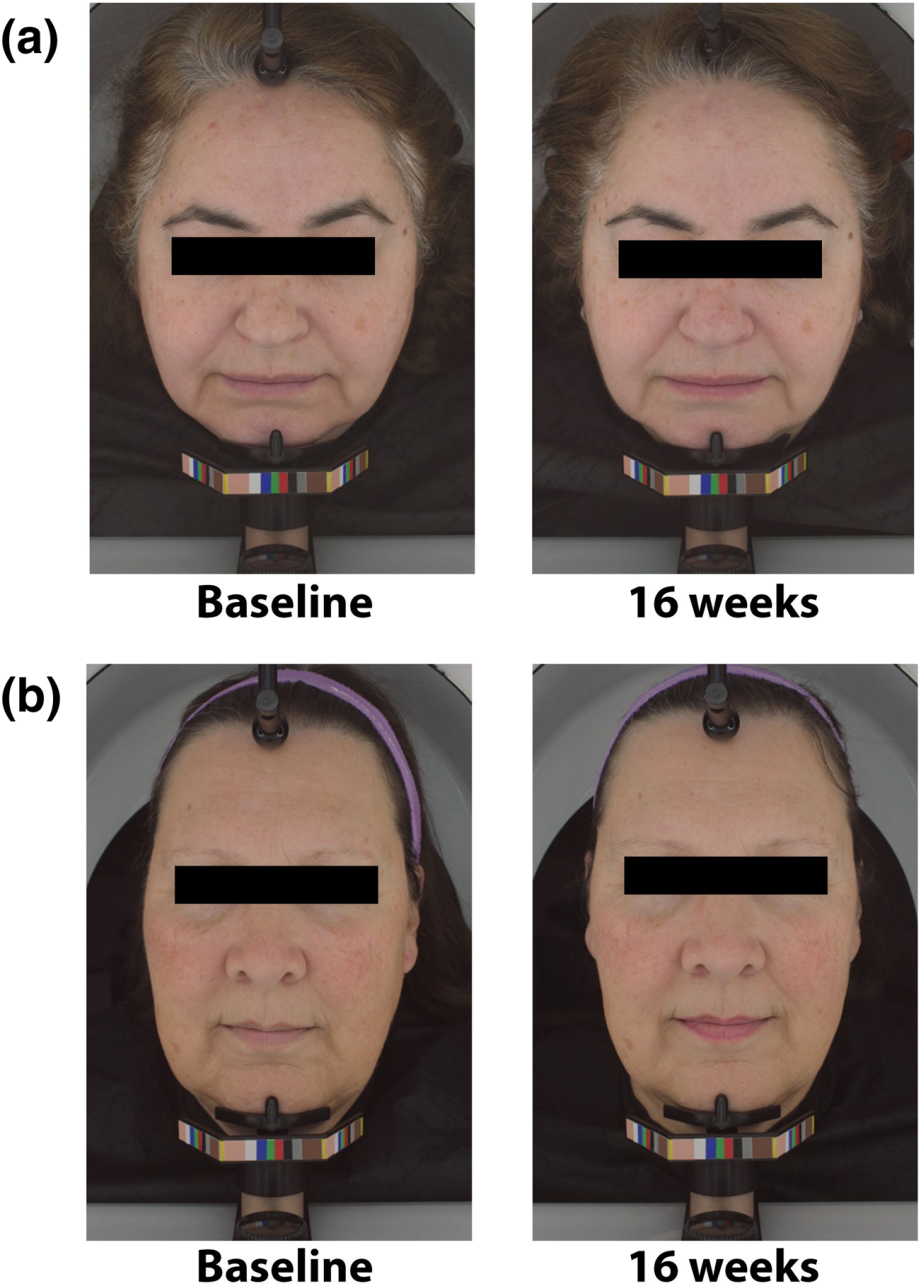
(a) Control subject and (b) almond subject facial images at baseline and after 16 weeks of receiving the almond or snack interventions, respectively [Colour figure can be viewed at http://wileyonlinelibrary.com]

Our randomized, investigator‐blinded clinical trial demonstrates that daily consumption of almonds significantly decreased wrinkle severity and wrinkle width in postmenopausal females. See Figure 4 for facial images of control group and almond group subjects at baseline and after 16 weeks of almond consumption. In particular, for consumers who value food‐based therapies as nutritional options for antiaging purposes, almond consumption has appeal due to its overall health‐promoting benefits including essential fatty acids and vitamin E. Of particular interest is that almonds have benefits beyond the skin‐related effects noted in this study (Hyson, Schneeman, & Davis, 2002; Kalita et al., 2018). For instance, one clinical study demonstrated that in patients with coronary artery disease who consumed a small dose (10 g/day) of almonds that were soaked overnight and taken on an empty stomach, the levels of the protective high‐density lipoprotein was significantly increased in addition to improvement in other lipid parameters after 6 and 12 weeks (Jamshed, Sultan, Iqbal, & Gilani, 2015).

Research has previously demonstrated that sebum excretion rate is negatively associated with wrinkle severity (Foolad et al., 2015). Our results show that wrinkle severity can be improved even when the sebum excretion remains unchanged. Moreover, our findings suggest that sebum excretion rate may not present the full story and that an assessment of the sebum lipid profile is warranted. An assessment of the lipid profile and vitamin E levels is planned for future studies.

Nutrition as a prominent factor in aging processes is gaining recognition as scientific research emerges establishing the role of specific foods and supplements in skin health. Almonds provide approximately 12.5 mg vitamin E (or alpha‐tocopherol) per 50.0‐g serving (National Nutrient Database for Standard Reference Release 28: Basic report, Almonds, 2016). Vitamin E is a fat‐soluble vitamin with strong antioxidant properties that is incorporated into cell membranes and effectively protects against free radical damage caused by intrinsic and exogenous stressors including ultraviolet radiation (Keen & Hassan, 2016; Tanyel & Mancano, 1997). When ingested, dietary alpha‐tocopherol can increase skin concentrations of vitamin E, whereas synthetic versions of alpha‐tocopherol have not demonstrated this effect (Richelle, Sabatier, Steiling, & Williamson, 2006). In another study, fibroblasts taken from elderly individuals incubated with vitamin E demonstrated a decrease in activity of collagen degradation enzymes called matrix metalloproteinases, and thus, vitamin E may prevent collagen degradation (Ricciarelli, Maroni, Ozer, Zingg, & Azzi, 1999). Extrapolated to a clinical setting, dietary vitamin E from the consumption of almonds may serve natural antiaging benefits. Alterations in serum fatty acid profile as a result of almond ingestion may also play a role in our results of significantly decreased wrinkle width and severity seen in this group, although we cannot confirm that with the present data. Previous work has demonstrated that higher intake of omega‐3 fatty acids from plant sources is significantly inversely associated with signs of photoaging (Latreille et al., 2013). Further studies of the link between dietary fatty acid ingestion and modulation of serum lipids are under ongoing and continuing investigation in future studies.

We utilized whole almonds in this study, but previous work has suggested that soaked or blanched almonds may have enhanced vitamin E content (Arslan, Ahmed, & Gilani, 2017). We did not blanch or soak almonds for practical reasons so that it was easier to prepare the almonds for the subjects without adding further variability to the almonds. However, future studies may consider comparing blanched or soaked almonds with whole almonds.

We used a relatively large dose (approximately 59 g/day) of almonds in this study, whereas a study on cardioprotective effects used a smaller dose of overnight soaked almonds (10 g/day) (Jamshed et al., 2015). Hence, future studies could explore lower doses of almonds when studying its effects on the skin. Although vitamin E consumed through food sources such as nuts and legumes has not shown toxicity, it is important to note that vitamin E supplementation in cumulative doses excess of 1,000 IU/day can inhibit clotting functions in the blood and interact with medications (Thiele, Hsieh, & Ekanayake‐Mudiyanselage, 2005). No toxicities were noted in our study.

### 4.1 Limitations

Because aging is a long‐lasting process, our 16‐week experimental findings can be difficult to generalize to extended periods of time. Second, skin aging is multifactorial in nature, and although we excluded people with a long or recent smoking history, there is a variance in participant‐aging confounders such as frequency of UV light exposure and emotional stress, which were outside the scope of our study. This study served as a pilot study with a limited number of subjects that were enrolled. Future studies will need to expand to a larger recruitment pool. Our study is limited to cosmetic evaluation, as no measurements were made regarding collagen production. Lastly, our study did not evaluate diseases or younger subjects; therefore, our findings are limited to otherwise healthy postmenopausal females.

## 5 CONCLUSIONS

Overall, our results suggest that daily consumption of almonds may be an effective option to prevent progression of normal aging including wrinkles in postmenopausal females. Our results warrant future studies with expanded population groups and additional evaluations for signs of skin aging.

## CONFLICT OF INTEREST

R.K.S. serves as a scientific advisor to Learn Health and consultant for Burt's Bees, Dermala, and Tomorrow's Leaf. N. F., A. R. V., I. R., W. B., G. M. C., J. W. N., and F. M. S. have no other conflicts of interest to report.
